# Analysis of the Genetic Diversity of *Houttuynia cordata* Thunb Germplasm and Associated Variations in Lead Content

**DOI:** 10.1002/pld3.70095

**Published:** 2025-08-07

**Authors:** Min He, Feifeng Mao, Liyu Wang, Guidong Zu, Jingwei Li, Xiuhong Xu, Wanping Zhang

**Affiliations:** ^1^ Vegetable Industry Research Institute of Guizhou University Guiyang China; ^2^ Agricultural College of Guizhou University Guiyang China; ^3^ Department of Agriculture and Rural Affairs of Guizhou Province Guiyang China; ^4^ Agricultural and Rural Bureau of Qiannan Buyei and Miao Autonomous Prefecture Duyun China; ^5^ Agriculture and Rural Bureau of Weining Yi, Hui and Miao Autonomous County Bijie China

**Keywords:** genetic diversity, *Houttuynia cordata*, ISSR markers, Pb^2+^ enrichment

## Abstract

*Houttuynia cordata* is an important medicinal and vegetable crop in Southwest China. Due to the accumulation of heavy metal ions such as lead ions (Pb^2+^) in *H. cordata* , consumption of this plant carries risks, such as ingestion of lead‐contaminated *H. cordata* , may lead to Pb^2+^ bioaccumulation, which is associated with developmental retardation, endocrine disruption, and impairments to immune and neurological functions. In order to screen *H. cordata* germplasm for low Pb^2+^ absorption and identify the Pb^2+^ adsorption‐related agronomic traits and molecular markers, the genetic diversity of a germplasm resource of *H. cordata* comprising collected 72 accessions was comprehensively evaluated based on agronomic traits and Pb^2+^ contents in the underground stems of the plant. Further, intersimple sequence repeats (ISSR) markers and generalized linear model (GLM) correlation analyses were performed to identify the ISSR loci related to the Pb^2+^ absorption. Ward clustering analysis grouped the 72 accessions of *H. cordata* into five major classes through analysis of morphological traits. Combined analysis of the Pb^2+^ contents of underground stems with the phenotypic traits revealed significant changes in members within the five classes, indicating that the Pb^2+^ content significantly affected the results of the evaluation of agronomic traits. Greyscale analysis and subsequent verification revealed that the underground stem thickness was closely related to the Pb^2+^ absorption. Based on ISSR markers and subsequent verification, two loci, namely, Locus 21 and Locus 29, were found to have better screening effects on germplasms with low Pb^2+^ adsorption. The accessions that did not carry these two loci in the genome generally exhibited low lead ion adsorption. This study presents a faster marker‐based screening for *H. cordata* plants that are safer for consumption.

## 1 Introduction

*Houttuynia cordata* Thunb. is a plant of the genus Houttuynia of the family Sambucus. This plant is included in the Chinese Pharmacopeia as a medicinal and edible plant. *H. cordata* can effectively inhibit viruses, reduce inflammation, and possess antibacterial, antioxidant, and anticancer potency (Wang et al. 2023; Rafiq et al. 2022; Wu et al. 2021; Adhikari et al. 2021; Du et al. 2021). In some parts of Asia, especially in Southwest China, *H. cordata* is widely used as a vegetable crop (Zhu et al. 2020). The roots of *H. cordata* are considered edible (Qi et al. 2022). In addition, *H.* *cordata* is also used in fermented beverages, health products, and the cosmetic industry (Wei et al. 2024).

*H. cordata* has a long cultivation history and serves as a great resource in China (Rafiq et al. 2022). Genetic and environmental factors together determine the crop agronomic traits, and accurate investigation of agronomic traits contributes to elite variety breeding and germplasm innovation (Cao et al. 2016; Jia et al. 2013). In previous studies by Yang et al. (2010), Qian et al. (2020), Chen et al. (2021), Li et al. (2023), and Zhao et al. (2023), the agronomic traits of germplasms of *Artemisia annua* , *Apium graveolens* , *Triticum aestivum* , *Gastrodia elata*, and *A. argyi* were successfully classified by cluster analysis, through which excellent performing genotypes were selected. Several special stress‐resistance traits, for instance, the heat tolerance of *A. graveolens* , were identified (Li et al. 2023). Thus, in this study, species clustering analysis was performed based on agronomic traits of *H. cordata* in order to identify from the germplasm accessions with better agronomic traits to serve as materials for breeding and efficient cultivation.

*H. cordata* has significant enrichment of heavy metals content that includes lead, chromium, mercury, and cadmium ions (Wang et al. 2018; Wang et al. 2020; Chen et al. 2019). The plant is particularly inclined to adsorb lead ions (Pb^2+^) (Zuo et al. 2022). Pb^2+^ exists in a variety of forms, with natural sources of Pb^2+^ comprising ores and unnatural sources such as industrial activities and transportation (Binh et al. 2021). Pb^2+^ poisoning can occur to varying degrees when humans live in Pb^2+^‐contaminated environments (Balali‐mood et al. 2021). Crops grown in Pb^2+^‐contaminated soils pose a safety risk to the production of agricultural products, increasing the risk of Pb^2+^ poisoning in humans (Bouida et al. 2022). Pb^2+^ poisoning can induce damage to the liver, kidneys, endocrine, immune, and neurological systems (García‐Niño and Pedraza‐Chaverrí 2014; Matović et al. 2015; Edelstein and Ben‐Hur 2018; Renu et al. 2021; Parida and Patel 2023). Pb^2+^ accumulation mainly occurs in the root system of *H. cordata* . The tolerance concentration to Pb^2+^ is up to 1000 mg kg^−1^ (Liu et al. 2018). Pb^2+^ enrichment results in a high dietary risk of exposure when consuming *H. cordata* . However, the main edible part of the plant is the roots. Thus, screening genotypes with low Pb^2+^ adsorption from abundant germplasm resources and analyzing the agronomic traits most related to Pb^2+^ adsorption can provide a basis for screening safe *H. cordata* varieties for consumption.

Molecular marker‐assisted breeding is an important method that combines modern molecular biology with traditional genetic breeding to develop more superior plant varieties (Duan et al. 2020; Chu et al. 2021). At present, intersimple sequence repeats (ISSR) marker is one of the main DNA markers used in plant breeding (Run et al. 2020). The ISSR method can produce a large number of polymorphic fragments at a relatively low cost (Amiteye 2021) and has been successfully applied to *Withania somnifera* , *Auricularia cornea*, and *Hordeum vulgare* , leading to the identification of molecular markers for seed weight (Khabiya et al. 2024; Du et al. 2024; Yigider et al. 2024). ISSR markers facilitate the selection of new varieties (Kücük et al. 2024).

In this study, cluster analysis of the agronomic traits of *H. cordata* was performed to obtain classification results based on agronomic characteristics. The effect of Pb^2+^ on the clustering of agronomic traits was studied based on the groupings obtained, and a genetic relationship map was constructed based on ISSR markers. A correlation analysis between ISSR markers and Pb^2+^ contents was carried out to identify the Pb^2+^ adsorption sites. The ultimate goal of this research was to provide a faster marker‐based screening guide for the breeding of high‐quality *H. cordata* with low Pb^2+^ adsorption for plants that are safer for consumption.

## 2 Materials and Methods

### 2.1 Experimental Materials

A germplasm resource of *H. cordata* comprising collected 72 accessions was evaluated in this study. The accessions were collected from 11 provinces and cities, including Guizhou Province (47 accessions), Sichuan Province (4 accessions), Yunnan Province (3 accessions), Chongqing City (3 accessions), Hunan Province (6 accessions), Hubei Province (4 accessions), Guangxi Province (1 accession), Jiangxi Province (1 accession), Fujian Province (1 accession), Anhui Province (1 accession), and Zhejiang Province (1 accession).

### 2.2 Planting of Materials

From February to April 2022, the *H. cordata* materials conserved at the Vegetable Research Institute of Guizhou University were transplanted in a farm located in Guiyang City for observation of the agronomic traits. The materials were incubated in 9 cm × 9 cm × 15 cm pots containing a planting substrate composed of peat: vermiculite: perlite = 3: 1: 1. In June 2022, the 72 *H. cordata* seedlings were planted in a substrate supplemented with 0 mg kg^−1^ or 500 mg kg^−1^ of lead nitrate [Pb (NO_3_)_2_, TMRM, China]. When preparing the contaminated matrix, a prepared Pb^2+^ solution at the specific concentration was added to the air‐dried soil that had been weighed (3 kg/basin). Then the solution was evenly poured into the basin. The substrate was then air‐dried before water was added. This process was repeated for 40 days to establish natural passivation with 500 mg kg^−1^ of the Pb^2+^ pollution matrix. At least four *H. cordata* roots were planted in each pot; each root contained three nodes (6 cm in length). Each treatment was repeated in three pots, and the experiment was repeated three times. The roots were cultivated and incubated in an artificial intelligence climate room at 25°C under a 16 h/8 h light/dark photoperiod. All materials were managed with a conventional cultivation method for 180 days.

### 2.3 Measurement of Morphological Traits

Based on the study by Guan (2010), the leaf edge traits, petiole color, leaf vein color, leaf edge color, leaf color, back color, stem color, and leaf shape of *H. cordata* were evaluated. The details of the morphological traits have been presented in Table 1. The height of the stem (cm), thickness of the stem above ground (mm), underground stem thickness (mm), crown diameter (cm), internode length (cm), leaf length (cm), and leaf width (cm) were determined by an electronic vernier caliper (Dexia, Shanghai, China) and band tape (ARL96027, Shanghai M&G Stationery Inc., China). At least five pots of each material were measured. The trait frequency is expressed as the percentage of accessions possessing the trait out of the total 72 accessions.

**TABLE 1:** Evaluation criteria for the quality traits of *H. cordata* .

Indicator	Assignment criteria
Leaf edge shape	1, entire‐margined; 2, micro shrinkage; 3, waves
Petiole color	1, green; 2, light red; 3, red; 4, purple
Leaf vein color	1, green; 2, red
Leaf edge color	1, green; 2, red
Leaf color	1, light green; 2, green; 3, dark green
Leaf dorsal color	1, green; 2, light red; 3, red; 4, purple
Stem color	1, green; 2, light red; 3, red; 4, purple
Leaf type	1, short heart; 2, long heart

### 2.4 Pb^2+^ Treatment and Content Measurement

*H. cordata* materials treated with 0 mg kg^−1^ and 500 mg kg^−1^ of Pb^2+^ for 180 days were washed with tap water for 10 min and further washed with deionized water three times for 10 min each. The materials were then dried and crushed. The content of Pb^2+^ in *H. cordata* was determined by inductively coupled plasma mass spectrometry (ICP‐MS) according to Paul et al. (2021).

### 2.5 DNA Extraction and ISSR Reaction

DNA extraction was conducted according to Zhu et al. (2012). The primer sequences used in this experiment were obtained from the ninth set of ISSR primers published by Columbia University in Canada and Fan (2015). A total of eight primers with clear bands were screened out for testing and statistical analysis. The primer numbers and sequences are shown in Table 2.

**TABLE 2:** ISSR primer sequences and sources.

No.	Sequences	Source
13	ACACACACACACACACGC	Fan (2015)
18	AGAGAGAGAGAGAGAGGG	
834	AGAGAGAGAGAGAGAGYT	UBC Primer Set#9
841	GAGAGAGAGAGAGAGAYC	
866	CTCCTCCTCCTCCTCCTC	
879	CTTCACTTCACTTCA	
888	BDBCACACACACACACA	
900	ACTTCCCCACAGGTTAACACA	

*Note:* B = G, C or T; D = A, G or T; Y = C or T.

The total reaction volume was 20 μL, with 2 μL of template DNA, 1 μL of primer, 10 μL of 2xTaq enzyme, and 7 μL of ddH_2_O. The polymerase chain reaction (PCR) procedure was as follows: predenaturation to 94°C for 3 min, followed by cycles of denaturation to 94°C for 30 s, annealing to 54°C for 1 min, and extension to 72°C for 2 min for a total of 35 cycles. The PCR products were then kept at 72°C for 10 min. The amplified products were separated by electrophoresis on 1.5% agarose gel. They were then observed and photographed on a ChampChemi610 (BeijingSaizhi, China).

### 2.6 Data Analysis

The standard deviation and coefficient of variation of *H. cordata* were calculated by Excel (Microsoft, USA). Principal components and correlation analysis of the agronomic traits were performed using IBM SPSS 26 (International Business Machines Corporation, USA). Cluster analysis of the agronomic traits was carried out using Origin 2021 (OriginLab, Japan), and cluster analysis of the ISSR results was carried out using NTSYS 2.10e (Applied Biosystems, USA). Using Tassel 4.0 (Cornell University, USA), the joint analysis of the Pb^2+^ content and ISSR molecular markers in 72 *H. cordata* accessions was performed under a generalized linear model (GLM). The interpretation rate (*R* ^2^) of each marker site and the corresponding phenotypic variation at a significance threshold of *p* < 0.01 were obtained.

## 3 Results

### 3.1 Analysis of the Qualitative Traits of *H. cordata* Accessions

**FIGURE 1:**
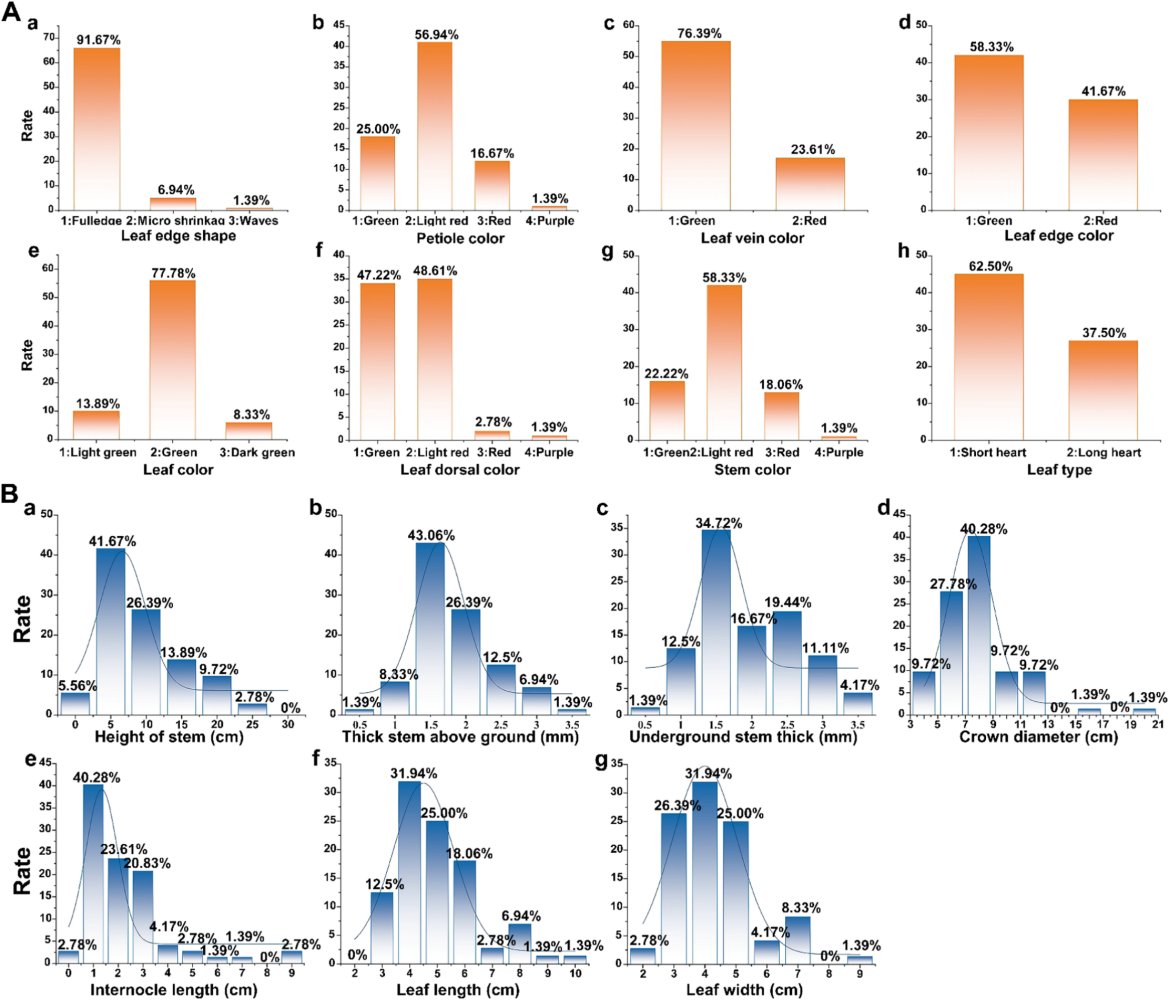
Cluster analysis of the agronomic traits of 72 *H. cordata* accessions. (A): qualitative traits; (B): quantitative traits. (a) in A: leaf edge shape; (b) in A: petiole color; (c) in A: leaf vein color; (d) in A: leaf edge color; (e) in A: leaf color; (f) in A: leaf dorsal color; (g) in A: stem color; (h) in A: leaf type. (a) in B: height of stem; (b) in B: thickness of stem above ground; (c) in B: underground stem thickness; (d) in B: crown diameter; (e) in B: internode length; (f) in B: leaf length; (g) in B: leaf width.

Among the 72 accessions of *H. cordata* , 91.67% of leaves were entire‐margined. The petiole color was mainly light red (56.94%), while purple stems accounted for only 1.39% of resources. Further, 76.39% of leaf veins were green, while 23.61% of leaf veins had a red vein color. There were three leaf colors: green was the most common (77.78%) and dark green was the least common (8.33%). The leaf dorsal color was mainly green and light red, accounting for 47.22% and 48.61%, respectively. The stem color was mainly light red (58.33%); purple was rare (1.39%). Of the two leaf edge colors, green was predominant, accounting for 58.33% of 72 accessions. Among the 72 *H. cordata* accessions, most had leaves with a short heart shape (62.50%); the remaining accessions had a long heart shape (Figure 1A).

### 3.2 Analysis of the Quantitative Traits of *H. cordata* Accessions

The stem height was divided into six categories with a range of 5 cm. The majority of the accessions making up the studied germplasm resource had a stem height of 2.5–7.5 cm (41.67%); a stem height of 22.5–27.5 cm accounted for only 2.78% of resources. Aboveground stem diameter was divided into seven categories with a range of 0.5 mm: an aboveground stem diameter of 1.25–1.75 mm was the most common, accounting for 43.06% of the total *H. cordata* germplasm resource; aboveground stem diameters of 0.25–0.75 mm and 3.25–3.75 mm were the least common, accounting for 1.39% of the 72 accessions, respectively. The rhizome diameter was divided into seven categories with a 0.5 mm interval between each category. A rhizome diameter of 1.25–1.75 mm was the most common (34.72%), while one of 0.25–0.75 mm was the least common (1.39%). The crown width was divided into nine categories with a range of 2 cm. The 7–9 cm group was the most common, accounting for 40.28% of the 72 accessions, while the 19–21 cm group was the least common, accounting for 1.39% of the 72 accessions. The length of the internodes was divided into 10 categories with an interval of 1 cm. The most common was the 0.5–1.5 cm group, accounting for 40.28% of the 72 accessions. The 5.5–6.5 cm and 6.5–7.5 cm groups were the least common, accounting for 1.39% of all accessions, respectively. The leaf length was divided into eight categories with an interval of 1 cm. Among them, the 3.5–4.5 cm category was the most common, accounting for 31.94% of the total resources, and the 8.5–9.5 cm category and 9.5–10.5 cm category were the least common, accounting for 1.39% of all the accessions, respectively. The width of the leaves was divided into eight categories, with an interval of 1 cm between each category. Among them, the 3.5–4.5 cm category was the most common, accounting for 31.94% of the 72 accessions, and the 8.5–9.5 cm group was the least common, accounting for 1.39% of all the accessions **(**Figure 1B **)**.

### 3.3 Cluster Analysis of the Morphological Traits of *H. cordata* Accessions

**FIGURE 2:**
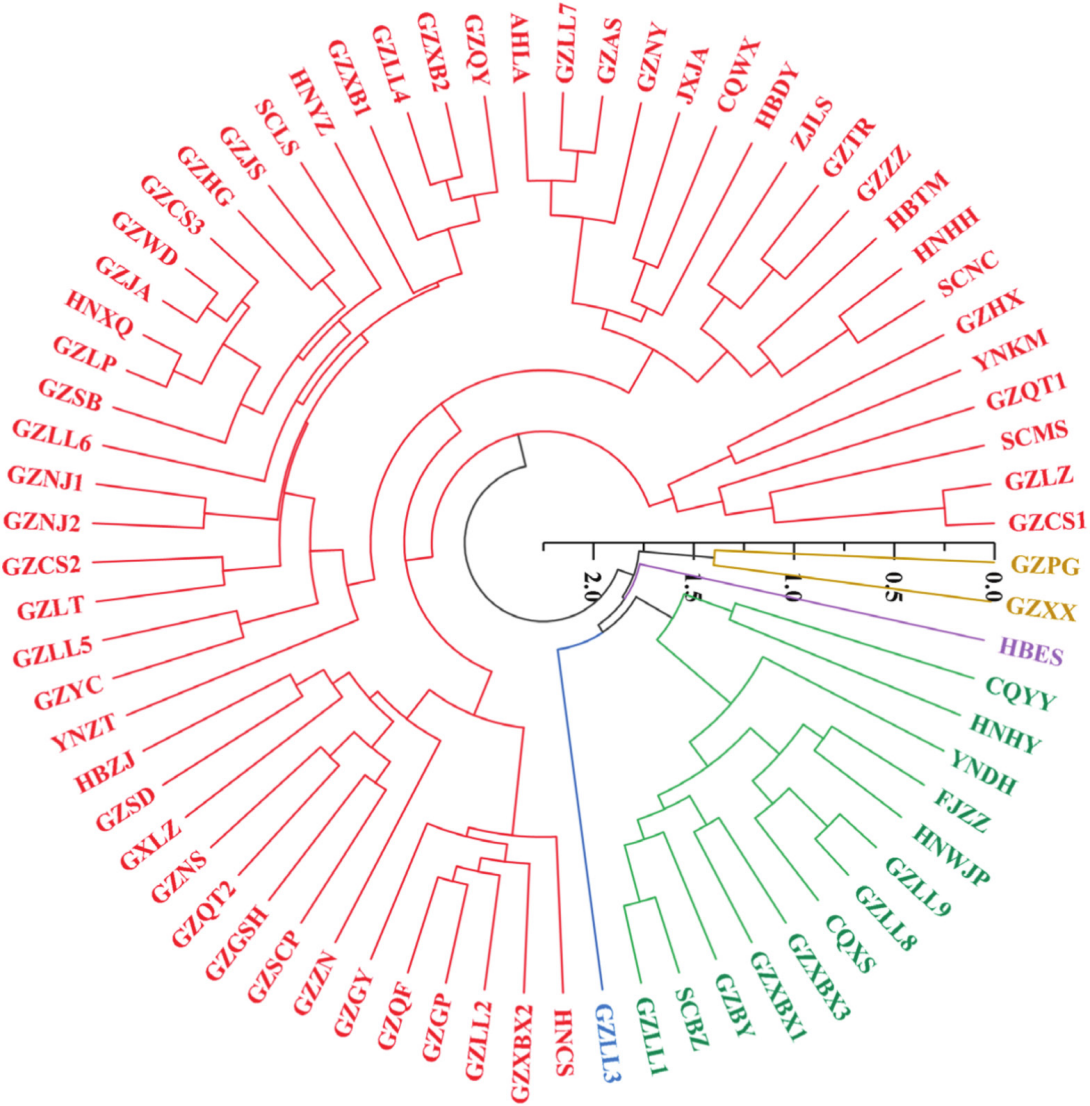
Cluster analysis of the morphological traits of 72 accessions of *H. cordata* .

The 72 accessions of *H. cordata* were divided into 5 categories. Class I included 55 accessions; among them, 38 accessions were collected from Guizhou Province, 4 from Hunan Province, 3 from Sichuan Province, 3 from Hubei Province, and 2 from Yunnan. The remaining germplasms came from Chongqing City and Anhui, Jiangxi, Guangxi and Zhejiang Provinces, respectively. Each only accounted for 1 piece of plant material. Subfamily I of this cluster was composed of 6 accessions, and subfamily II was composed of 49 accessions. Class II was composed of 13 accessions, including 6 accessions from Guizhou Province, 2 from Chongqing, 2 from Hunan, and 1 each fromYunnan, Fujian and Sichuan, respectively. Class II was divided into 2 subfamilies; subfamily I of cluster II included 2 accessions, and subfamily II included 11 accessions. Class III consisted of GZPG and GZXX. Class IV contained only one accession, HBES. Accessions GZLL3 was classified into class V (Figure 2).

### 3.4 Cluster Analysis of Pb^2+^ Enrichment in *H. cordata* Accessions

**FIGURE 3:**
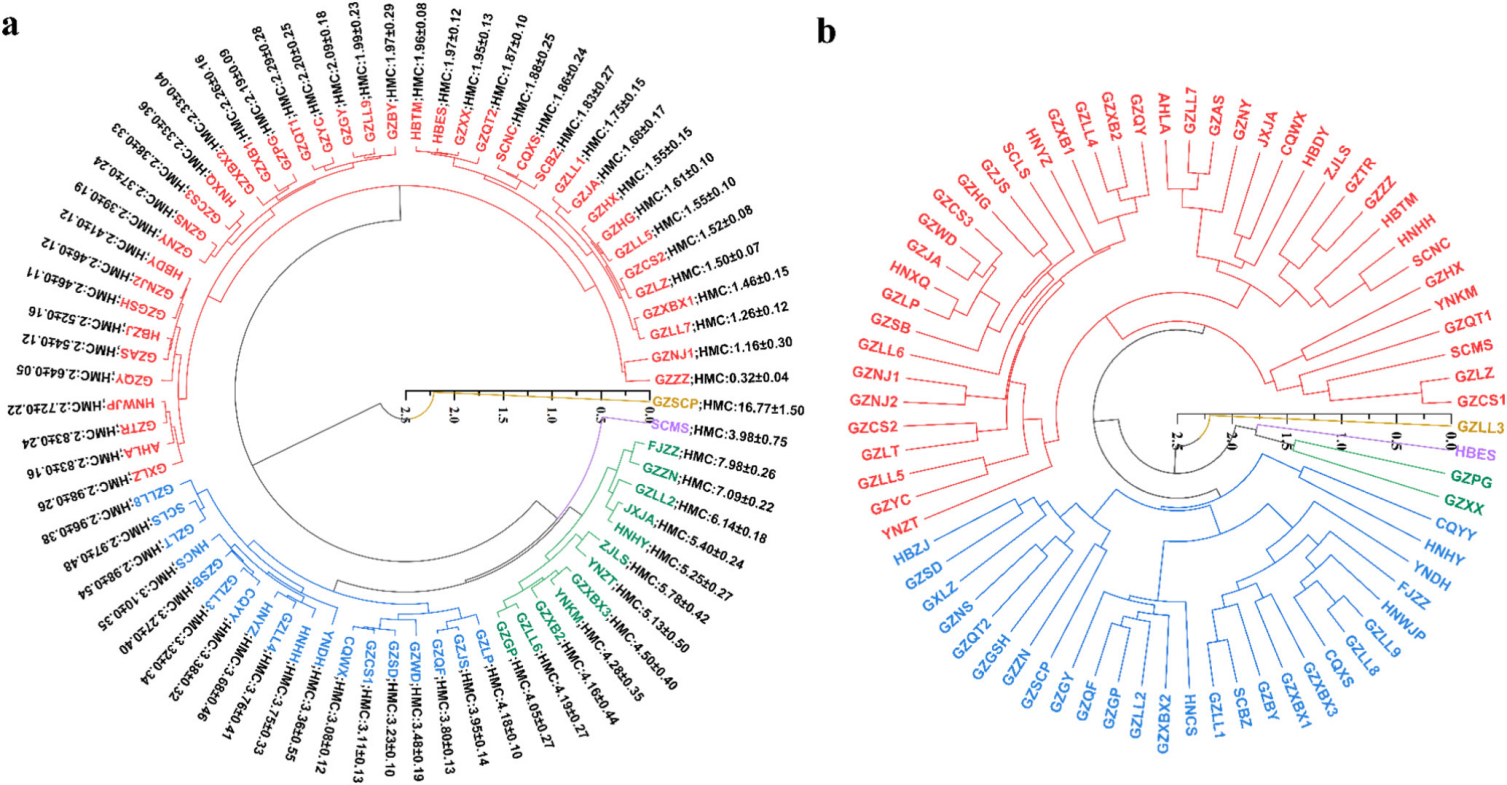
Cluster analysis of the Pb^2+^ enrichment ability. (a) Cluster analysis of agronomic traits and endogenous Pb^2+^ content. (b) Cluster analysis of 72 accessions of *H. cordata* .

The ward cluster analysis grouped the 72 accessions of *H. cordata* into 5 categories according to the Pb^2+^ content of the roots of each genotype. The largest class included 40 accessions. Among them, 29 were collected from Guizhou, 4 from Hubei, 2 each from Sichuan and Hunan, 1 from Chongqing, and 1 each from Anhui and Guangxi. The Pb^2+^ enrichment ability ranged from 0.32 to 2.98 mg kg^−1^. Accessions GZNJ1 and GZZZ, with Pb^2+^ contents of 1.16 mg kg^−1^ and 0.32 mg kg^−1^, respectively, had the weakest Pb^2+^ enrichment abilities. The 40 accessions of Class I were further divided into 2 subfamilies comprising 22 and 18 accessions, respectively. The Pb^2+^ enrichment ability of the accessions comprising subfamily I ranged from 1.97 to 2.98 mg kg^−1^; for subfamily II, the Pb^2+^ enrichment ability ranged from 0.32 to 1.96 mg kg^−1^. Class II was comprised of 18 accessions. Among them, 11 were collected from Guizhou, 3 from Hunan, 2 from Chongqing, and 1 each from Yunnan and Sichuan. The Pb^2+^ enrichment ability ranged from 2.96 to 4.18 mg kg^−1^. Class III consisted of 12 accessions. Among them, 6 were collected from Guizhou, 2 from Yunnan, 1 from Fujian, 1from Jiangxi, and 1 each from Hunan and Zhejiang. The Pb^2+^ enrichment ability ranged from 4.05 to 7.98 mg kg^−1^. Only 1 accession was enriched in class IV, SCMS, with an enrichment ability of 3.98 mg kg^−1^. Only 1 accession was enriched in class V, GZSCP. This had the highest endogenous Pb^2+^ enrichment of 16.77 mg kg^−1^ (Figure 3a).

Using both the morphological traits and endogenous Pb^2+^ content as variables, ward cluster analysis classified *H. cordata* 72 accessions into 5 categories. Class I comprised 41 accessions, including 27 from Guizhou, 3 each from Sichuan and Hunan, 2 each from Yunnan and Hubei, and 1 each from Chongqing, Anhui, Jiangxi, and Zhejiang. Subfamily I was composed of accessions GZCS1, GZLZ, SCMS, GZQT1, YNKM, and GZHX, and subfamily II comprised 35 accessions. There were 27 accessions of Class II, among which 17 accessions were collected from Guizhou, 3 from Hunan, 2 from Chongqing, and 1 each from Yunnan, Sichuan, Hubei, Guangxi, and Fujian. Class II was divided into two subfamilies; subfamily I included CQYY and HNHY, and subfamily II included 25 accessions. Class III consisted of GZPG and GZXX. Class IV comprised only 1 accession, HBES. AccessionsGZLL3 clustered separately into class V (Figure 3b).

### 3.5 Greyscale Analysis Between the Morphological Indices and Pb^2+^ Content of *H. cordata* Accessions

A gray correlation analysis among 15 morphological traits and Pb^2+^ content was carried out. The crown width, diameter of the aboveground stem, leaf dorsal color, diameter of the underground stem, and leaf edge shape were the five indices with the highest correlations with the Pb^2+^ content; all correlations were above 0.900. The petiole color, leaf edge color, stem color, leaf color, and shape of the leaf were the five characteristics with the weakest correlations with the Pb^2+^ content, with correlations ranging from 0.792 to 0.870 (Table 3).

**TABLE 3:** Gray correlation analysis of the morphological traits and Pb^2+^ content of *H. cordata* .

Evaluation items	Correlation	Rank
Crown width	0.907	1
Diameter of the aboveground stem	0.907	2
Leaf dorsal color	0.905	3
Diameter of the undergound stem	0.902	4
Leaf edge shape	0.900	5
Leaf length	0.899	6
Leaf width	0.897	7
Leaf vein color	0.884	8
Height of the stem	0.881	9
Length of the stem node	0.881	10
Shape of the leaf	0.870	11
Leaf color	0.862	12
Stem color	0.830	13
Leaf edge color	0.792	14
Petiole color	0.792	15

Among the five indices that have the strongest correlations with the Pb^2+^ content in *H. cordata* , only the diameter of the underground stem exhibited an inverse correlation with Pb^2+^ absorption. The thicker the underground stem, the smaller the surface area in contact with the soil per unit soil volume, and the less likely it is for the stem to absorb Pb^2+^; the thinner the stem, the larger the surface area in contact with the soil, and the more Pb^2+^ it can absorb. Among all the accessions in this study, those with a stem diameter greater than 2.50 ± 1.04 mm had Pb^2+^ ≤ 3 mg kg^−1^, while those with a stem diameter less than 2.17 ± 0.68 mm had a significantly increased Pb^2+^ content, which had Pb^2+^ > 3 mg kg^−1^. Based on the morphological index data collected in the present study, there were no significant relationships between the absorption of Pb^2+^ and other morphological indices such as the crown diameter, diameter of the aboveground stem, leaf dorsal color, and leaf edge shape (Table 4).

**TABLE 4:** Verification analysis of the most relevant morphological traits of Pb^2+^ absorption in *H. cordata* based on the greyscale analysis results.

Pb^2+^ content (mg kg^−1^)	Crown diameter (cm)	Diameter of the stem above ground (mm)	Leaf dorsal color	Diameter of the stem underground (mm)	Leaf edge shape
0.00 < Pb^2+^ ≤ 1.00	7.25 ± 1.65 b	1.93 ± 0.42 ab	1.00 ± 0.00 ab	3.17 ± 0.30 a	1.00 ± 0.00 a
1.00 < Pb^2+^ ≤ 2.00	9.20 ± 0.20 ab	1.94 ± 0.43 ab	1.00 ± 0.00 ab	2.38 ± 1.08 ab	1.00 ± 0.00 a
2.00 < Pb^2+^ ≤ 3.00	8.67 ± 0.12 ab	2.38 ± 0.66 ab	1.33 ± 0.58 a	2.50 ± 1.04 ab	1.33 ± 0.58 a
3.00 < Pb^2+^ ≤ 4.00	9.57 ± 0.40 ab	1.90 ± 0.33 ab	1.00 ± 0.00 ab	1.63 ± 0.37 b	1.00 ± 0.00 a
4.00 < Pb^2+^ ≤ 5.00	7.50 ± 1.59 b	1.80 ± 0.36 b	1.00 ± 0.00 ab	1.79 ± 0.31 b	1.00 ± 0.00 a
5.00 < Pb^2+^ ≤ 6.00	9.03 ± 1.12 ab	1.85 ± 0.48 ab	1.00 ± 0.00 ab	2.17 ± 0.68 ab	1.00 ± 0.00 a
6.00 < Pb^2+^ ≤ 7.00	10.68 ± 0.07 a	2.18 ± 0.01 ab	1.00 ± 0.00 b	1.65 ± 0.01 b	1.00 ± 0.00 a
7.00 < Pb^2+^ ≤ 8.00	8.50 ± 2.50 ab	1.88 ± 0.30 ab	1.00 ± 0.00 ab	1.65 ± 0.36 b	1.00 ± 0.00 a
Pb^2+^> 8.00	9.21 ± 0.02 ab	2.58 ± 0.01 a	1.00 ± 0.00 b	1.74 ± 0.04 b	1.00 ± 0.00 a

### 3.6 Correlation Analysis Between ISSR Markers and Pb^2+^ Content in *H. cordata* Accessions

**FIGURE 4:**
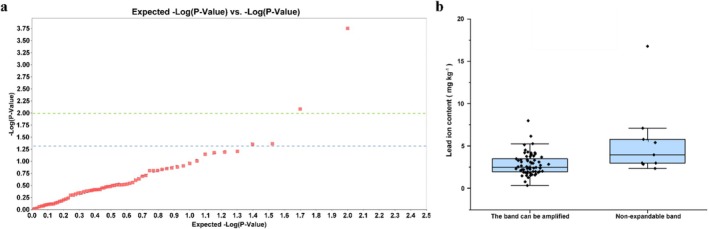
Correlation analysis between the Pb^2+^ content and ISSR markers in 72 *H. cordata* accessions. (a) Analysis of loci that were significantly associated with the Pb^2+^ content, the green line and blue line indicate *p* = 0.01 and *p* = 0.05, respectively. Panel (b) indicates the relationship between the Pb^2+^ content of accessions and lSSR locus existence.

Eight ISSR primers were used to amplify 72 *H. cordata* accessions. The results were as follows: A total of 100 bands were amplified, with an average of 12.5 bands per primer, and 99 bands were polymorphic; the average number of amplified polymorphic bands was 12.38. The total number of bands produced by ISSR primers ranged from 11 to a maximum of 15, with an average polymorphism rate of 99.04%; the homology of origin of different populations was proved (Table 5). GLM analysis of the Pb^2+^ content and ISSR markers showed that four loci were associated with the Pb^2+^ content at *p* < 0.05. Among them, two loci, Locus 19 and Locus 95, were found at *P* < 0.01, with corresponding phenotypic explanatory rates of 18.31% and 9.53%, respectively (Figure 4a and Table 6). The two loci were found in nine accessions (Figure 4b and Table 6).

**TABLE 5:** Results of locus detection by ISSR marker.

Name of primer	Primer sequences	Total number of strips	Number of polymorphic bands	Polymorphism ratio
13	ACACACACACACACACGC	15	15	100
18	AGAGAGAGAGAGAGAGGG	11	11	100
834	AGAGAGAGAGAGAGAGYT	12	12	100
841	GAGAGAGAGAGAGAGAYC	13	13	100
866	CTCCTCCTCCTCCTCCTC	11	11	100
879	CTTCACTTCACTTCA	12	12	100
888	BDBCACACACACACACA	13	12	92.31
900	ACTTCCCCACAGGTTAACACA	13	13	100
Total Average		100 12.5	99 12.38	99.04

*Note:* B = G. C. T; D = A. G. T; Y=C.T.

**TABLE 6:** Four ISSR marker loci were significantly associated with the Pb^2+^ content of 72 *H. cordata* accessions.

Trait	Locus	Marker_F	marker_P	markerR^2^
Pb^2+^ content	19	15.6912	0.0002	0.1831
Pb^2+^ content	95	7.3798	0.0083	0.0954
Pb^2+^ content	21	4.1896	0.0444	0.0565
Pb^2+^ content	76	4.2293	0.0435	0.0570

The five accessions with the lowest Pb^2+^ contents and five accessions with the highest Pb^2+^ contents were selected from the total of 72 accessions and used as validation materials. Among the four ISSR loci significantly associated with Pb^2+^ absorption, Locus 19 could not be amplified in accessions with low Pb^2+^ adsorption, while amplicons were obtained in some accessions with high Pb^2+^ adsorption. Amplicons of Locus 95 were obtained in all high adsorption accessions, while in their low Pb^2+^ adsorption counterparts, Locus 95 was detected in two accessions but not in three accessions. Locus 21 was similar to Locus 19, but the number of high Pb^2+^ adsorption accessions s in which this locus could be detected was less than for Locus 19. Locus 76, in contrast to Locus 19 and Locus 21, was detected in all five low Pb^2+^ adsorption accessions; among the high Pb^2+^ adsorption accessions, this locus was detected in three but could not be detected in two accessions. Accessions containing ISSR markers of Locus 19, Locus 95, and Locus 21 may have strong adsorption capacity for Pb^2+^, while Locus 19 and Locus 21 had better screening effects on accessions with low Pb^2+^ adsorption; the accessions that did not carry these two loci in the genome were generally low lead ion adsorption accessions. Accessions without Locus 76 had higher Pb^2+^ adsorption, but those containing Locus 76 had high or low Pb^2+^ adsorption (Table 7).

**TABLE 7:** Validation analysis of the Pb^2+^ concentration and existence of Pb^2+^‐related ISSR loci in *H. cordata* .

Accessions	Pb^2+^ content	Locus existence			
		Locus 19	Locus 95	Locus 21	Locus 76
GZZZ	0.32 ± 0.04	0	0	0	1
GZCS2	1.16 ± 0.30	0	0	0	1
GZLL7	1.26 ± 0.12	0	1	0	1
GZXBX1	1.46 ± 0.15	0	0	0	1
GZLZ	1.50 ± 0.07	0	1	0	1
ZJLS	5.78 ± 0.42	1	1	1	1
GZLL2	6.14 ± 0.18	0	1	0	1
GZZN	7.09 ± 0.22	1	1	1	0
FJZZ	7.98 ± 0.26	0	1	0	1
GZSCP	16.77 ± 1.50	1	1	0	0

## 4 Discussion

The analysis of genetic diversity in agronomic characteristics in collected accessions plays an important role in studies of germplasm resource utilization. Field agronomic trait studies and clustering analysis have been widely applied in the screening of crop germplasms with desired features and have uncovered relationships between traits and crop characteristics. For instance, Zhu et al. (2023), Weldemichael et al. (2023), He et al. (2024) and Zuo et al. (2024) analyzed crop germplasms and the agronomic traits of chili ( *Capsicum annuum* ), sesame ( *Sesamum indicum* ), coix ( *Coix lacryma‐jobi* ), and sweet potato ( *Dioscorea esculenta* ). Using cluster analyses, Zhu et al. (2023) discovered that chili varieties with high levels of capsaicin have smaller fruit types, thinner flesh, smaller single fruit weights, and upright stem types. Weldemichael et al. (2023) found that high‐quality sesame exhibits a short‐stem shape and lodging resistance. He et al. (2024) obtained eight high‐quality coix varieties through the analysis of agronomic traits such as the plant height, seed setting rate, effective tiller number, and stem thickness. Zuo et al. (2024) analyzed 8 agronomic traits of 15 sweet potato varieties and obtained three high‐quality varieties. In this study, the researchers demonstrated that stem diameter, number of branches per plant, and commodity rate were positively correlated with the selection of high‐quality varieties.

*H. cordata* is considered a niche vegetable, with its consumers mainly located in the southwestern region of China. Therefore, research on the agronomic traits of its germplasm resources is limited. Although Guan (2010) conducted a study of the agronomic traits of *H. cordata* , there is a lack of a comprehensive in‐depth genetic analysis of the agronomic traits of *H. cordata* . In this study, clustering analysis of the agronomic traits related to the morphology of *H. cordata* was performed in 72 accessions, and results show a clustering of the accessions into five classes (Figure 2). After adding the root Pb^2+^ content as an indicator to the clustering analysis, significant changes were observed in the accessions comprising the five classes (Figure 3). For example, in the agronomic trait clustering with only the morphological indicators, Class I comprised 55 accessions and Class II comprised 13 accessions. When the lead content was added to the clustering analysis, Class I was reduced to 14 accessions and Class II was increased to 27 accessions, which reflected the sum of the Class II accessions based solely on the morphological indicators plus the reduced accessions in Class I (Figure 3). The results of this study are similar to those of Huang et al. (2023) who conducted a cluster analysis of the agronomic traits of radish ( *Raphanus sativus* ). However, the current results are inconsistent with those of clustering analyses conducted with agronomic traits when clubroot resistance characteristics were supplemented. When crop agronomic traits are statistically analyzed, studies usually focus only on morphological and yield indicators (Vetriventhan et al. 2024), with a lack of attention to physiological characteristics. The results of the current study demonstrate that when conducting agronomic trait analysis; it is necessary to comprehensively consider physiological characteristic indicators in the analysis process.

Pb^2+^ is a heavy metal ion that can cause significant harm to the human body. Elevated blood lead levels lead to brain oedema, encephalopathy, mental confusion, drowsiness, coma, epilepsy, and even death (Yang et al. 2020). Many studies have shown that *H. cordata* is enriched in Pb^2+^ (Zeng 2007; Wu and Wu 2008; Wu et al. 2011). Gray correlation analysis between the agronomic traits and Pb^2+^ content was conducted, and the crown width, aboveground stem diameter, leaf dorsal color, underground stem diameter, and leaf edge shape were found to be the five most relevant indicators of Pb^2+^ adsorption (Table 3). Subsequently, correlation verification was conducted on the five indicators and Pb^2+^ adsorption, and a negative correlation between the underground stem diameter and lead absorption was found (Table 4). This may be because the thinner the underground stem, the larger the surface area of the stem in contact with the soil per unit volume and weight, and thus, the higher the rate of absorption of Pb^2+^. However, further evidence is needed to support this hypothesis. In subsequent research and applications in agricultural production, the diameter of the underground stem could serve as a reference indicator for the selection of *H. cordata* accessions with different Pb^2+^ absorption abilities.

Four Pb^2+^ content‐related ISSR markers were identified by GLM analysis. Analysis of the associations between Pb^2+^ and molecular markers is helpful for identifying Pb^2+^ absorption‐related loci in *H. cordata* and can provide a basis for marker‐assisted molecular breeding of *H. cordata* varieties with low adsorption of Pb^2+^ (Table 6, Figure 4). Among the four ISSR markers, Locus 19 and Locus 21 exhibited better screening effects on accessions with low Pb^2+^ adsorption; the screening effects of the other two markers on the adsorption of Pb^2+^ in accessions were not very significant (Table 7). Thus, in subsequent research, more in‐depth functional validation should be conducted on the genes of Locus 19 and Locus 21 to further examine their ability to control the adsorption of lead ions in *H. cordata* .

In this study, 72 *H. cordata* accessions were classified into five classes by clustering analysis of agronomic traits and Pb^2+^ content. The thickness of the underground stem exhibited the strongest correlation with the lead ion content in the gray analysis. The average polymorphism rate of 72 *H. cordata* accessions was 99.04% according to ISSR marker analysis. The correlations between the ISSR markers and Pb^2+^ content were analyzed, and Locus 19 and Locus 21 were selected. These loci appear to be beneficial for screening high‐quality *H. cordata* accessions with low Pb^2+^ contents. These findings offer a faster basis for the molecular breeding of edible *H. cordata* varieties with significantly low lead ion content that are safer for consumption.

## Author Contributions

**Min He:** data curation, formal analysis, investigation, writing – riginal draft, review. **Feifeng Mao:** investigation. **Liyu Wang:** investigation. **Guidong Zu:** investigation. **Jingwei Li:** investigation, methodology, software. **Xiuhong Xu:** methodology, investigation. **Wanping Zhang:** conceptualization, data curation, formal analysis, investigation, project administration, writing – original draft, review, and editing.

## Ethics Statement

The authors have nothing to report.

## Consent

The authors have nothing to report.

## Conflicts of Interest

The authors declare no conflicts of interest.

## Peer Review

The peer review history for this article is available in the Supporting Information for this article.

## Supporting information


**Data S1.** Peer Review.

## Data Availability

The authors have nothing to report.
