# Supplementary material for: Analysis of the Genetic Diversity of Houttuynia cordata Thunb Germplasm and Associated Variations in Lead Content
Source: Plant Direct. 2025 Aug 7;9(8):e70095. doi: 10.1002/pld3.70095 (PMC12331442; doi:10.1002/pld3.70095)
Supplement: Supplementary file 1 — Data S1. Peer Review. [file PLD3-9-e70095-s001.pdf]

Plant Direct
Open Access

Analysis of the genetic diversity of Houttuynia cordata Thunb germplasm and its correlations with the Lead content

Wanping Zhang, Min He , Feifeng Mao , Liyu Wang , Guidong Zu , Jingwei Li , Xiuhong Xu

Decision letter Round 1

June 19, 2025

Ms. Wanping Zhang

Guizhou University Agricultural College

Guiyang, N/A

China

RE: Analysis of the genetic diversity of Houttuynia cordata Thunb germplasm and its correlations with the Lead content

Dear Wanping:

Thank you for submitting to Plant Direct. All required reviews have been returned and we have now finished our evaluation of your manuscript. In light of the reviewers' and editor's comments, further revisions are needed before the paper can be accepted for publication in Plant Direct.

Please view the editors' and reviewers' comments below and use their suggestions as a guide while you work on your revision.

When uploading the revised version of this article, please be sure to include the following:

-A word document that contains your response to the editor/reviewers. You should respond to each reviewer comment and note the changes made to the manuscript. If you do not agree with a reviewer's comment and choose not to make a suggested revision, please explain why. Please try to provide as complete an answer as possible to each reviewer's criticisms in the "response to reviewers" section.

Plant Direct
Open Access

-A tracked changes document

- A clean version of the latest version of the manuscript

To upload your revision, please click the link below.

https://plantdirect.msubmit.net/cgi-bin/main.plex?el=A5Lr4oO3A5pNf5I4A9ftdTeh69fmMyriKY45OGXQ0gwZ

In order to provide as timely a service as possible, we ask that your revision is resubmitted within 4-6 weeks after receipt of this request. If an extension is needed, please send a request, along with a brief explanation, to the editorial office at plantdirect@wiley.com .

Thank you very much for giving us an opportunity to review your work. I look forward to receiving the next version.

Sincerely,

Jonathan Ingram

------------------------------------------------------------------------ Editor comments:

I was very concerned to see the large no. of references listed but not cited, as noted by the reviewer. Can you explain the situation which led to this and reassure during revision that a full, careful check has been carried out.

Generally, please take the revision opportunity to check the manuscript content meticulously to ensure any remaining issues such as this are fully addressed.

Plant Direct

Open Access

------------------------------------------------------------------------------- Reviewer comments:

Reviewer #1:

1. Corrections and suggestions have been indicated throughout the manuscript and tracked to enable you improve the manuscript.

2. Many references listed in the references section have not been cited in the manuscript text.

3. The following references were cited bu

t not listed at the references section:

Run et al. 2020

Ventriventhan et al. 2024

Yang et al. 2020

Decision letter Round 2

July 4, 2025

Ms. Wanping Zhang

Guizhou University Agricultural College

Guiyang, N/A

China

MSID: 2025-01778R1

MS TITLE: Analysis of the genetic diversity of Houttuynia cordata Thunb germplasm and its correlations with the Lead content

Dear Ms. Zhang:

I am pleased to inform you that your manuscript "Analysis of the genetic diversity of Houttuynia cordata Thunb germplasm and its correlations with the Lead content" has been accepted for publication in Plant Direct.

Plant Direct
Open Access

Your article will appear online in the next available issue of Plant Direct. To ensure your article gets published as quickly as possible, please pay attention to the steps detailed below. We have found that most of the delays happen at this stage, especially at the payment stage, so please respond as quickly as possible when prompted.

License Agreement: Once your article has been accepted it will move to Production and undergo admin and file checking - you may receive an email with any queries we have at this stage. When all required items are received by the publisher and queries resolved, the corresponding author will receive an email from Wiley's Author Services system which will ask them to log on at https://authorservices.wiley.com/bauthor and will present them with the appropriate license for completion. Your article cannot be published until both the signed license agreement and payment of the article fee have been received.

Payment of the Open Access Article Publication Fee: All articles published in Plant Direct are fully open access: immediately and freely available to read, download and share. Plant Direct charges a publication fee to cover the publication costs. The corresponding author for this manuscript should have already received a quote from the payments team (cs-openaccess@wiley.com) with the estimated article publication fee; please email cs-openaccess@wiley.com if this has not been received. The corresponding author should log on to the Wiley Author Services site, where the publication fee can be paid by credit card or an invoice. Pro Forma can also be requested. Payment of the publication charge must be received before the article will be published online.

Proofs: You will have the opportunity to look over your paper once more when you receive the author proofs for your article. The proofs will be with you in approximately two weeks. Please note that, in addition to publishing reviewer comments, the author's responses to review comments will also be published alongside the final version of the paper. If you would not like the author's responses to be published, please contact the editorial office at plantdirect@wiley.com .

Promotion of your article: You can help your research get the attention it deserves! Check out Wiley's free Promotion Guide for best-practice recommendations for promoting your work at www.wileyauthors.com/eeo/guide. And learn more about Wiley Editing Services which offers professional video, design, and writing services to create shareable video abstracts, infographics, conference posters, lay summaries, and research news stories for your research at www.wileyauthors.com/eeo/promotion.

Thank you again for your contribution to Plant Direct. If you have any questions, feel free to contact the editorial office at plantdirect@wiley.com .

Sincerely,

Jonathan Ingram

Editor, Plant Direct

Response letter Round 1

Dear Editor,

Thank you for giving us the opportunity to submit a revised version of our Manuscript ID 2025-01778 entitled " Analysis of the genetic diversity of Houttuynia cordata Thunb germplasm and its correlations with the Lead content" for publication in the Plant Direct.

We appreciate the time and effort that you and the reviewers dedicated to providing feedback on our manuscript and are grateful for the insightful comments on and valuable improvements to our paper. We have incorporated most of the suggestions made by the reviewers. Those changes are highlighted in the revised manuscript. the changes section of manuscript has been highlighted with the track changes feature to visually show any changes we made to manuscript. Please see below, in red, for a point–by–point response to the reviewers' comments and concerns. All page numbers refer to the revised manuscript file with tracked changes.

We hope that our manuscript could be considered for publication in your journal.

Thank you very much for your help. If we need further modification, please do not hesitate to contact us, this will be a good opportunity for us to continue to learn and improve.

Best wishes,

Sincerely yours

Wanping Zhang

Email: 1226190368@qq.com

Response to Reviewer 1 Comments

Dear Reviewer 1,

On behalf of all the contributing authors, I would like to express our sincere appreciations of reviewer' professional comments concerning our article entitled "Analysis of the genetic diversity of Houttuynia cordata Thunb germplasm and its correlations with the Lead content" (Manuscript ID 2025-01778).

These comments are all valuable and helpful for improving the quality of our manuscript. According to the reviewer' comments, we have made modifications to our previous manuscript, the changes section of manuscript has been highlighted with the tracked changes feature to visually show any changes we made to manuscript. Please see below, in red, for a point–by–point response to the reviewer' comments and concerns. All page numbers refer to the revised manuscript file with tracked changes. the detailed revisions are listed below.

Thank you very much for your patient guidance and help. If we need further modification, please do not hesitate to contact us, this will be a good opportunity for us to continue to learn and improve.

Best wishes,

Sincerely yours

Reviewer 1

Thanks to the Editor and anonymous Reviewers for their comments on our paper. we have modified the manuscript to more rigorous standards. We have thoroughly addressed all grammatical and writing issues in the tracked-change version of the manuscript, with all modifications highlighted in red. In response to the reviewers' comments, we have supplemented the relevant content in the manuscript, which appears as highlighted text in the tracked-change document. The point-by-point responses to the Reviewers are listed below.

Comment 1: Indicate some of the risks associated with consumption of the plant.

Response: Thank you for raising this important point. In the revised manuscript, We have added the following sentence "such as ingestion of lead-contaminated H. cordata may lead to $Pb^{2+}$ bioaccumulation, which is associated with developmental retardation, endocrine disruption, and impairments to immune and neurological

functions”, This statement has been incorporated in Lines 25-27 of the revised manuscript, with the relevant text highlighted for easy reference.

Comment 2: Present ISSR and GLM in full.

Response: Thank you for this constructive suggestion. We have incorporated the full terminology as suggested, with the following additions in the manuscript “inter-simple sequence repeats (ISSR) markers and generalized linear model (GLM)”, The full terms have been added in Line 31 of the tracked-changes manuscript, as highlighted in the revised version.

Comment 3: Why is work described as a theoretical basis of screening? The work presents a faster marker based practical screening for safer plants.

Response: Thank you for this insightful observation. We agree that the original phrasing ("theoretical basis of screening") did not fully capture the practical innovation of our marker-based approach. In the revised manuscript, We have revised the terminology to “This study presents a faster marker-based screening for H. cordata plants that are safer for consumption.” The corresponding modification is located in Lines 41-42 of the tracked-changes manuscript.

Comment 4: It is not clear how this percentage was obtained. This should be clarified in the Materials and Methods section.

Response: Thank you for highlighting this important methodological point. We have revised the Materials and Methods section to provide a detailed explanation of the percentage derivation process。We have added the following sentence “The trait frequency is expressed as the percentage of accessions possessing the trait out of the total 72 accessions.” The corresponding modification is located in Lines 128-129 of the tracked-changes manuscript.

Comment 5: Was this percentage based on the 72 accessions or based on number of leaves?

Response: Thank you for this clarification request. The percentage values reported in our study were calculated exclusively based on the 72 accessions (not individual leaves). We have revised the terminology from "leaves" to "accessions" throughout

the manuscript to maintain consistency with the germplasm-based analysis. The corresponding modification is located in Lines 169 of the tracked-changes manuscript.

Comment 6: Indicate the value or the range.

Response: Thank you for prompting this clarification. We have added the requested numerical values/ranges in the revised manuscript. We have added the following sentence “which had Pb2+>3 mg kg-1

.” The corresponding modification is located in

Lines 265-266 of the tracked-changes manuscript.

Comment 7: Not cited in text.

Response: We sincerely apologize for this citation omission. We have now properly cited the previously omitted reference in the appropriate sections of the manuscript. The corresponding modification is located in Lines 49 of the tracked-changes manuscript.

Comment 8: Not cited in text.

Response: Thank you for your careful review. Upon reevaluation, we confirm that the uncited reference (Alsafran, M., K. Usman, B. Ahmed, M. Rizwan, M. H. Saleem, and H. Al Jabri. 2022. “Understanding 388387 the phytoremediation mechanisms of potentially toxic elements: a proteomic overview of recent 389388 advances.” Frontiers in Plant Science 13: 881242.) is not directly relevant to our current study. We have now removed the uncited reference from the manuscript.

Comment 9: Not cited in text.

Response: We sincerely apologize for this citation omission. We have now properly cited the previously omitted reference in the appropriate sections of the manuscript. The corresponding modification is located in Lines 50 of the tracked-changes manuscript.

Comment 10: Not cited in text.

Response: We sincerely apologize for this citation omission. We have now properly cited the previously omitted reference in the appropriate sections of the

manuscript. The corresponding modification is located in Lines 74 of the tracked-changes manuscript.

Comment 11: Not cited in text.

Response: Thank you for your careful review. Upon reevaluation, we confirm that the uncited reference (Fuller, R., P. J Landrigan, K. Balakrishnan, G. Bathan, S. Bose-O'Reilly, M. Brauer, J. Caravanos, T. Chiles, A. Cohen, L. Corra, M. Cropper, G. Ferraro, J. Hanna, D. Hanrahan, H. Hu, D. Hunter, G. Janata, R. Kupka, B. Lanphear, M. Lichtveld, K. Martin, A. Mustapha, E. Sanchez-Triana, K. Sandilya, L. Schaefli, J. Shaw, J. Seddon, W. Suk, M. M. Téllez-Rojo, and C. H. Yan. 2022. “Pollution and health: a progress update.” The Lancet. Planetary Health 6: e535-e547.) is not directly relevant to our current study. We have now removed the uncited reference from the manuscript.

Comment 12: Not cited in text.

Response: We sincerely apologize for this citation omission. We have now properly cited the previously omitted reference in the appropriate sections of the manuscript. The corresponding modification is located in Lines 73 of the tracked-changes manuscript.

Comment 13: Not cited in text.

Response: Thank you for your careful review. Upon reevaluation, we confirm that the uncited reference (Hou, S. Y., X. M. Ren, Y. Yang, D. H. Wang, W. Du, X. F. Wang, H. Y. Li, Y. H. Han, L. L. Liu, and Z. X. Sun.2022. “Genome-wide development of polymorphic microsatellite markers and association analysis of major agronomic traits in core germplasm resources of Tartary Buckwheat.” Frontiers in Plant Science 13: 819008.) is not directly relevant to our current study. We have now removed the uncited reference from the manuscript.

Comment 14: Not cited in text.

Response: Thank you for your careful review. Upon reevaluation, we confirm that the uncited reference (Kushwaha, A., N. Hans, S. Kumar, and R. Rani. 2018. “A critical review on speciation, mobilization and toxicity of lead in soil-microbe-plant system

and bioremediation strategies. ” Ecotoxicology and Environmental Safety 147: 1035-1045.) is not directly relevant to our current study. We have now removed the uncited reference from the manuscript.

Comment 15: Not cited in text.

Response: Thank you for your careful review. Upon reevaluation, we confirm that the uncited reference (Lei, M. L., X. Liu, Y. Z. Wang, X. J. Zhang, H. L. Du, and Z. X. Mu. 2023. “Evaluation of local wheat (Triticum aestivum L.) germplasm resources and mining of excellent drought resistance loci in Shanxi province.” Genetic Resources and Crop Evolution 70: 1097-1113.) is not directly relevant to our current study. We have now removed the uncited reference from the manuscript.

Comment 16: Not cited in text.

Response: Thank you for your careful review. Upon reevaluation, we confirm that the uncited reference (Liu, X. C., J. Tian, Z. R. Zhou, Y. Z. Pan, and Z. Q. Li. 2023. “Antioxidant activity and interactions between whey protein and polysaccharides from different parts of Houttuynia cordata.” Frontiers in Nutrition 10: 1020328.) is not directly relevant to our current study. We have now removed the uncited reference from the manuscript.

Comment 17: Not cited in text.

Response: Thank you for your careful review. We appreciate your attention to detail and would like to clarify that the reference in question (Parida, L., and T. N. Patel. 2023. “Systemic impact of heavy metals and their role in cancer development: a review.” Environmental Monitoring and Assessment 195: 766.) was in fact cited in our original manuscript. For transparency, the corresponding modification is located in Lines 74 of the tracked-changes manuscript.

Comment 18: Not cited in text.

Response: Thank you for your careful review. Upon reevaluation, we confirm that the uncited reference (Peng, S. 2007. “Cloning of Houttuynia cordata HMGR gene fragment and ISSR analysis of genetic relationship.” Hunan University.) is not directly relevant to our current study. We have now removed the uncited reference

from the manuscript.

Comment 19: Not cited in text.

Response: Thank you for your careful review. Upon reevaluation, we confirm that the uncited reference (Plaza, P. I., M. Uhart, A. Caselli, G. Wiemeyer, and S. A. Lambertucci. 2018. “A review of lead contamination in South American birds: The need for more research and policy changes. ” Perspectives in Ecology and Conservation 16: 201-207.) is not directly relevant to our current study. We have now removed the uncited reference from the manuscript.

Comment 20: Not cited in text.

Response: Thank you for your careful review. Upon reevaluation, we confirm that the uncited reference (Poudel, K., A. Ikeda, H. Fukunaga, M. N. Brune Drisse, L. J. Onyon, J. Gorman, A. Laborde, and R. Kishi. 2023. “How does formal and informal industry contribute to lead exposure? A narrative review from Vietnam, Uruguay, and Malaysia.” Reviews on Environmental Health 2: 6.) is not directly relevant to our current study. We have now removed the uncited reference from the manuscript.

Comment 21: Not cited in text.

Response: Thank you for your careful review. Upon reevaluation, we confirm that the uncited reference (Tong, S. Y., Y. Wang, Z. N. Su, and D. L. Chen. 2023. “Evolution characteristics and influencing factors of population distribution in Southwest China since 2000.” Development of Small Cities and Towns 12: 13-21+44.) is not directly relevant to our current study. We have now removed the uncited reference from the manuscript.

Comment 22: Not cited in text.

Response: Thank you for your careful review. Upon reevaluation, we confirm that the uncited reference (Vasudevan, S., and M. A. Oturan. 2014. “Electrochemistry: as cause and cure in water pollution—an overview.” Environmental Chemistry Letters 12: 97-108.) is not directly relevant to our current study. We have now removed the uncited reference from the manuscript.

Comment 23: Not cited in text.

Response: Thank you for your careful review. Upon reevaluation, we confirm that the uncited reference (Wu, W., Y. L. Zheng, R. W. Yang, Z. H. Yan, and Y. M. Wei. 2003. "Analysis on genetic diversity of germplasm resources of cordate Houttuynia by ISSR marker." Wold Science and Technology/Modernization of Traditional Chinese Medicine and Materia Medica 5: 70-77.) is not directly relevant to our current study. We have now removed the uncited reference from the manuscript.

Comment 24: Not cited in text.

Response: Thank you for your careful review. Upon reevaluation, we confirm that the uncited reference (Xiao, S. Z., X. B. Dai, L. X. Zhao, Z. L. Zhou, L. K. Zhao, P. Xu, B. Q. Gao, A. Zhang, D. L. Zhao, R. Yuan, Y. Wang, J. Wang, Q. L. Li, and Q. H. Cao. 2023. "Resequencing of sweetpotato germplasm resources reveals key loci associated with multiple agronomic traits." Horticulture Research 10: uhac234.) is not directly relevant to our current study. We have now removed the uncited reference from the manuscript.

Comment 25: Not cited in text.

Response: Thank you for your careful review. Upon reevaluation, we confirm that the uncited reference (Zan FG, Zhang YB, Wu ZD, Zhao J, Wu CW, Zhao Y, Chen XK, Zhao LP, Qin W, Yao L, Xia HM, Zhao, P. F., K. Yang, J. Y. Liu, and X. P. Yang. 2020. "Genetic analysis of agronomic traits in elite sugarcane (Saccharum spp.) germplasm." PLoS One 15: e0233752.) is not directly relevant to our current study. We have now removed the uncited reference from the manuscript.

Comment 26: Not cited in text.

Response: Thank you for your careful review. We appreciate your attention to detail and would like to clarify that the reference in question (Zhao, C. X., J. C. Shen, S. K. Lv, F. H. Ye, S. X. Yin, M. S. Yang, R. J. Liu, D. M. Liu, H. G. Zhang, Y. H. Shen, and W. J. Chen. 2023. "Identification and evaluation of important agronomic traits in 44 Polish wheat varieties (Triticum polonicum L.) grown on the Qinghai Plateau, China." Plant Genetic Resources - Characterization and Utilization 21: 274-282.) was in fact cited in our original manuscript. For transparency, the corresponding

modification is located in Lines 58 of the tracked-changes manuscript.

Comment 27: Not cited in text.

Response: Thank you for your careful review. We appreciate your attention to detail and would like to clarify that the reference in question (Zhu, L., X. R. Xue, Z. X. Liang, L. Y. Xu, G. F. Yu, Y. Tang, and H. X. Li. 2023. “Correlation between capsaicin content and agronomic traits in pepper (Capsicum annuum L.) germplasm resources.” Acta Agriculturae Zhejianggensis 35: 862-872.) was in fact cited in our original manuscript. For transparency, the corresponding modification is located in Lines 318 of the tracked-changes manuscript.
